# The genome of *Litomosoides sigmodontis* illuminates the origins of Y chromosomes in filarial nematodes

**DOI:** 10.1371/journal.pgen.1011116

**Published:** 2024-01-16

**Authors:** Lewis Stevens, Manuela Kieninger, Brian Chan, Jonathan M. D. Wood, Pablo Gonzalez de la Rosa, Judith Allen, Mark Blaxter

**Affiliations:** 1 Tree of Life, Wellcome Sanger Institute, Cambridge, United Kingdom; 2 Lydia Becker Institute of Immunology and Inflammation, Wellcome Centre for Cell-Matrix Research, Faculty of Biology, Medicine & Health, University of Manchester, Manchester, United Kingdom; University of Georgia, UNITED STATES

## Abstract

Heteromorphic sex chromosomes are usually thought to have originated from a pair of autosomes that acquired a sex-determining locus and subsequently stopped recombining, leading to degeneration of the sex-limited chromosome. The majority of nematode species lack heteromorphic sex chromosomes and determine sex using an X-chromosome counting mechanism, with males being hemizygous for one or more X chromosomes (XX/X0). Some filarial nematode species, including important parasites of humans, have heteromorphic XX/XY karyotypes. It has been assumed that sex is determined by a Y-linked locus in these species. However, karyotypic analyses suggested that filarial Y chromosomes are derived from the unfused homologue of an autosome involved in an X-autosome fusion event. Here, we generated a chromosome-level reference genome for *Litomosoides sigmodontis*, a filarial nematode with the ancestral filarial karyotype and sex determination mechanism (XX/X0). By mapping the assembled chromosomes to the rhabditid nematode ancestral linkage (or Nigon) elements, we infer that the ancestral filarial X chromosome was the product of a fusion between NigonX (the ancestrally X-linked element) and NigonD (ancestrally autosomal). In the two filarial lineages with XY systems, there have been two independent X-autosome chromosome fusion events involving different autosomal Nigon elements. In both lineages, the region shared by the neo-X and neo-Y chromosomes is within the ancestrally autosomal portion of the X, confirming that the filarial Y chromosomes are derived from the unfused homologue of the autosome. Sex determination in XY filarial nematodes therefore likely continues to operate via the ancestral X-chromosome counting mechanism, rather than via a Y-linked sex-determining locus.

## Author summary

In many organisms, sex is determined by the presence or absence of a distinct, sex-limited chromosome (such as the Y chromosome in mammals). Nematodes, also known as roundworms, typically lack differentiated sex chromosomes and determine sex by a mechanism that counts the number of X chromosomes (with males having one X and females having two). However, several filarial nematode species, including important parasites of humans, have Y chromosomes that have often been assumed to play a role in sex determination. We assembled a high-quality genome sequence for *Litomosoides sigmodontis*, a rodent-infecting filarial nematode that lacks a Y chromosome. We show that filarial nematodes with Y chromosomes have undergone recent X-autosome fusion events and that the Y chromosomes are derived from the unfused copy of the autosome. We suggest that filarial Y chromosomes play no role in sex determination and that sex continues to be determined using the ancestral X-chromosome counting mechanism. The presence of X-autosome fusions in other nematodes that do not have Y chromosomes suggests that the filarial nematode Ys represent a snapshot of evolution in action: relict chromosomes on the path to eventual loss.

## Introduction

Genetic sex determination is often associated with the presence of heteromorphic sex chromosomes, where males and females differ in their karyotypes (e.g. XY versus XX). The sex-limited member of the heteromorphic chromosome pair often carries a primary sex determination locus, such as SRY on the Y chromosome of males in mammals [1,2]. The classical model of heteromorphic sex chromosome evolution posits that they originate from a pair of autosomes that acquire a sex-determining locus and subsequently stop recombining [3–5]. The lack of recombination in the sex-limited chromosomes (i.e. the Y or W) leads to degeneration. Sex-limited chromosomes tend to have fewer genes, accumulate repeats, and show extensive degeneration of coding loci, but are maintained because of the essential function of the sex determination loci they carry [4,6,7]. In contrast, many species have sex chromosomes but lack Y or W chromosomes. In these species, the heterogametic sex is haploid for the sex chromosome and sex is determined via a mechanism that assesses the autosome-to-sex chromosome ratio [8,9]. The most common system is XX/X0 (X-null) sex determination, where males have only one X and produce sperm that either carry or do not carry an X chromosome. In some lineages with XX/X0 systems, *de novo* evolution of a Y chromosome has resulted from an X-autosome fusion event (e.g. in grasshoppers [10–12]). In these cases, the unfused autosomal partner of the fused autosome behaves as a neo-Y chromosome, present only in males where it pairs with the formerly autosomal part of the extended X.

Filarial nematodes (from family Onchocercidae within suborder Spirurina [13]) are a group of animal-parasitic nematodes that cause a range of important human and veterinary diseases [14–16]. In contrast to most other nematodes, which typically have XX/X0 sex chromosome systems, several species of filarial nematode have XX/XY heteromorphic sex chromosome systems. Filarial XX/XY systems are proposed to have evolved twice independently from an ancestral XX/X0 system, once in the last common ancestor of *Dirofilaria* and *Onchocerca* and once in the last common ancestor of *Brugia* and *Wuchereria* [17–19]. It has often been assumed that sex determination in these species is driven by the presence or absence of a putative sex-determining locus present on the Y chromosome, by analogy to XY systems in other taxa (e.g. [20]). However, karyotype analyses found that the evolution of XY systems in both filarial lineages coincided with an X-autosome fusion, suggesting that the Y chromosome may be derived from the unfused homologue of the autosome involved in the fusion [17]. Sex determination in XY filarial nematodes may therefore operate similarly to species with XX/X0 systems, where sex is determined by a mechanism that counts the number of X chromosomes. In the free-living nematode *Caenorhabditis elegans*, where the mechanisms of sex determination have been well-studied, the number of X chromosomes is assessed through a set of X-linked loci termed X-signal elements (XSEs) [21]. The expression of the XSEs from the two copies of the X chromosome in *C*. *elegans* hermaphrodites (which are modified females capable of producing sperm) is sufficient to repress the activity of *xol-1*, the primary sex-determining gene. In contrast, XSE expression in males, which possess only a single X chromosome, is insufficient to suppress *xol-1*.

The increasing availability of chromosome-level reference genomes has led to a renewed focus on chromosome evolution. Using the genomes of extant species, the linkage groups present in the last common ancestor have been inferred for many taxa, including *Drosophila* [22], vertebrates [23], and Lepidoptera [24]. Seven ancestral linkage groups, called Nigon elements, are predicted to have been present in the last common ancestor of nematodes in the order Rhabditida [19,25]. The chromosomes of extant rhabditid species can be derived from combinations of these elements. For example, the *C*. *elegans* X chromosome is derived from a fusion between NigonX (the ancestrally X-linked Nigon element) and NigonN (ancestrally autosomal) that occurred in the last common ancestor of all *Caenorhabditis* species [19]. A recent analysis attempted to reinfer Nigon elements in an effort to understand the evolution of the filarial nematode XY sex chromosome systems, but erroneously inferred six ancestral rhabditid linkage groups instead of seven [20] (S1 Text). In doing so, the origins of the filarial Y chromosomes were obscured. In addition, the conflicting hypotheses regarding the number of chromosomes in the last common rhabditid ancestor have led some authors to avoid using the Nigon terminology, despite a seven-Nigon rhabditid ancestor being consistent with their data [26].

To clarify the chromosomal biology of the filarial nematodes, we generated a chromosome-level reference genome for the rodent-parasitic species *Litomosoides sigmodontis*, which has a 5A, XX/X0 karyotype [17] that is likely to match that of the ancestor of the Onchocercidae [19]. We show that the *L*. *sigmondontis* X chromosome is the product of an ancient fusion event between NigonX and NigonD that occurred in or prior to the last common ancestor of all filarial nematodes. By analysing chromosomally complete genome sequences and Nigon element assignments within a phylogenetic context, we confirm that the two lineages with XY sex chromosome systems have undergone two recent and independent X-autosome fusions involving different autosomes. We show that the homologous region between the neo-X and neo-Y chromosomes corresponds to the ancestrally autosomal portion of the neo-X chromosome, confirming that filarial neo-Y chromosomes are derived from the unfused homologues of the autosomes involved in the fusion events. We hypothesise that sex determination in filarial nematodes with XY sex chromosome systems operates similarly to the X-chromosome counting mechanism described in *C*. *elegans* and not by the presence of a sex-determining locus on the Y chromosome. The retention of the Y because of haploinsufficiency of the fused homologue in males is therefore likely to be an evolutionary intermediate to loss.

## Results

### *The* L. sigmodontis *X chromosome is a fusion of NigonX and NigonD*

**Fig 1:**
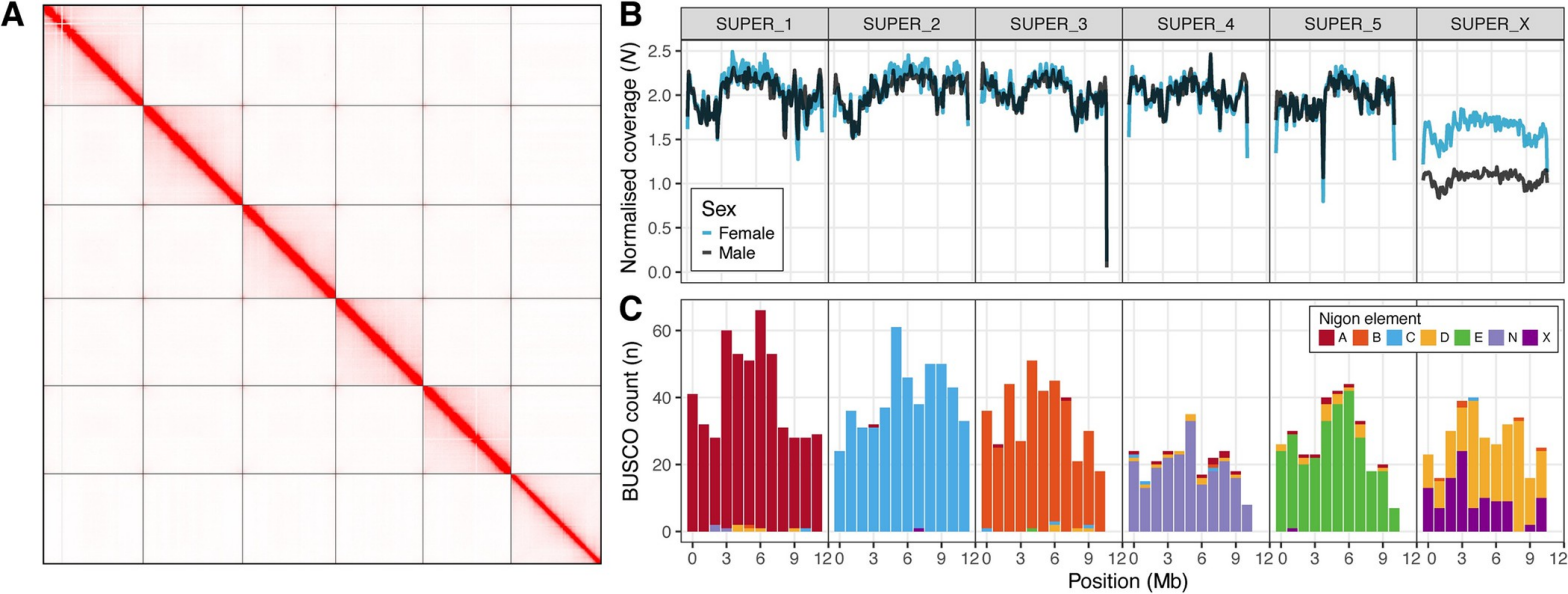
The *Litomosoides sigmodontis* X chromosome is a fusion of Nigon X and Nigon D. (A) Hi-C contact map for the nxLitSigm11.1 reference genome derived from the male Hi-C data. (B) Normalized male and female Hi-C read coverage in 100 kb windows in the six *L*. *sigmodontis* chromosomes. Normalised coverage (*N*) was calculated by dividing the coverage in each 100 kb window by the median autosomal coverage. (C) Distribution of counts of BUSCO genes in 500 kb windows in the six *L*. *sigmodontis* chromosomes coloured by their allocation to the seven Nigon elements (A-E, N, X).

We used a combination of high-coverage PacBio HiFi data derived from a single female and Hi-C data derived from two separate pools of adult males and females to generate a chromosome-level reference genome for *L*. *sigmodontis* (Fig 1A and Table 1). We scaffolded all of the 65.9 Mb primary nuclear genome assembly into six chromosome-sized scaffolds, consistent with the published *L*. *sigmodontis* karyotype (five autosomes plus the X chromosome) [17]. All six chromosomal scaffolds were similar in size (10.3–11.7 Mb) and were capped by arrays of the nematode telomeric repeat ([TTAGGC]_n_) at both ends (S1 Fig). The assembly is highly complete (96.0% using Benchmarking Using Single Copy Orthologues [BUSCOs] [27] with the Nematoda odb10 dataset) and has high base-level accuracy (QV of 52.9, corresponding to one error every 194 kb). We also generated a circular mitochondrial genome (spanning 13.7 kb) and a circular *w*Ls *Wolbachia* endosymbiont genome (spanning 1.05 Mb).

**Table 1:** Metrics for chromosome-level reference genome of *Litomosoides sigmodontis* and three other filarial nematode species.

***Accession information***				
Species	*Litomosoides sigmodontis*	*Dirofilaria immitis*	*Onchocerca volvulus*	*Brugia malayi*
BioProject	PRJEB64423	-	PRJEB513	PRJNA10729
Reference	This work	[28]	[29]	[20]
***Reference genome metrics***				
Span (Mb)	65.9	89.4	96.4	88.2
Scaffolds (n)	6 (+MT)	27	708	197
Scaffold N50 (Mb)	10.9	15.2	25.5	14.2
Span in chromosome-sized scaffolds (%)	100.0	98.6^3^	94.3	92.1
Contigs (n)^1^	28	167	1006	205
Contig N50 (Mb)^1^	5.0	4.2	1.0	10.3
BUSCO completeness (%)^2^	96.0	90.1	97.7	98.2
BUSCO duplication (%)^2^	0.4	0.5	0.9	0.6
QV	52.9	-	-	-
***Gene set metrics***				
Number of protein-coding genes	9,686	No gene set available	12,109	10,878
Number of transcripts	11,646	No gene set available	13,436	15,985
BUSCO completeness (%)^2^	95.7	No gene set available	98.2	98.9

1. Contig values were calculated by splitting scaffolds at ≥10 consecutive Ns.

2. Genome and gene set completeness was assessed using BUSCO (version 5.2.2) with the nematoda_odb10 dataset (using the Augustus option when assessing genome completeness).

3. The *D*. *immitis* genome was scaffolded to chromosomes by comparison to *O*. *volvulus* PRJEB513.

To identify the *L*. *sigmodontis* X chromosome, we aligned male and female Hi-C data to the genome and calculated per-base read coverage. Consistent with the reported karyotype, we found five scaffolds that had diploid coverage in both males and females and are thus identified as autosomes (Fig 1B). The remaining scaffold had haploid coverage in males and was identified as the X chromosome (Fig 1B). In females, this scaffold had approximately 80% of the coverage of the autosomal scaffolds (Fig 1B). We interpret this as being due to the fact that adult female *L*. *sigmodontis* carry large numbers of both male and female embryos retained *in utero*, and this results in sub-diploid coverage of the X.

We painted the autosomes and X chromosome using conserved BUSCO genes allocated to the seven Nigon elements [19]. The five autosomes were primarily composed of loci from a single Nigon element (NigonA, B, C, E, and N), suggesting that these chromosomes have not undergone fusion or fission since the last common rhabditid ancestor (Fig 1C). In contrast, the X chromosome was composed of genes from both NigonX and NigonD (Fig 1C). The NigonX and NigonD genes were highly intermixed, suggesting that sufficient evolutionary time has passed since the fusion event for intra-chromosomal rearrangements to have blended the distinct Nigon partitions.

### Filarial nematodes with XY sex chromosomes have undergone recent X-autosome fusions

**Fig 2:**
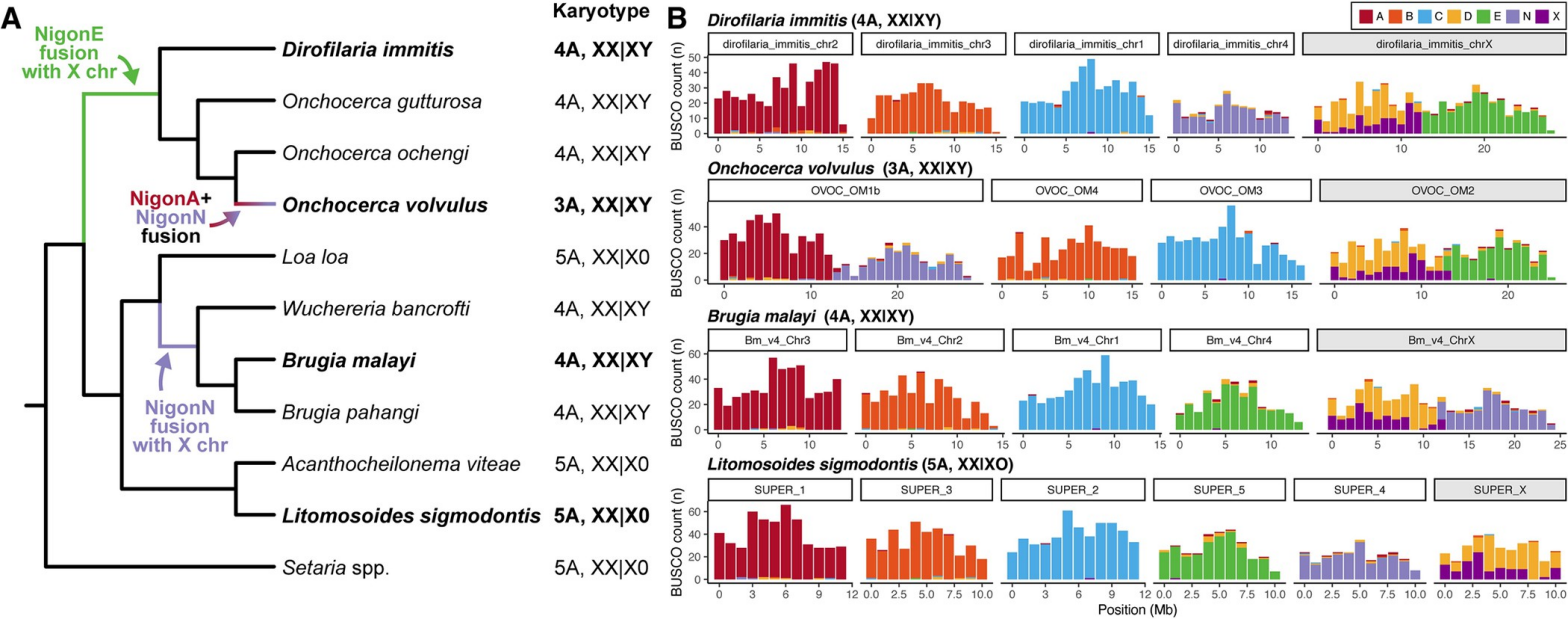
Filarial nematodes with XY sex chromosomes have undergone recent X-autosome fusions. (A) Cladogram of filarial nematodes derived from a supermatrix comprising 1,757 single-copy orthologues present in all 16 species. Reported karyotypes are indicated [17,30]. Note that the haploid chromosome number (*n*) in the genus *Setaria* varies from *n* = 3 to *n* = 6, but the ancestral state is believed to be *n* = 6 [17,30]. Species with chromosome-level reference genomes figured in (B) are highlighted in bold. (B) Distribution of BUSCO genes coloured by their allocation to the seven ancestral Nigon elements [19] in the chromosome-level assemblies of *Dirofilaria immitis*, *Onchocerca volvulus*, *Brugia malayi*, and *L*. *sigmodontis*. The scaffold that comprises the X chromosome is indicated in grey. Species names and karyotypes are indicated above each plot.

To understand the evolutionary origins of the filarial XY systems, we analysed previously reported karyotypes and Nigon assignments in three XY species with chromosome-level reference genomes (*Onchocerca volvulus* [29], *Brugia malayi* [20], and *Dirofilaria immitis* [28]), within the phylogenetic context of the Onchocercidae [18] (Fig 2). Post (2005) previously inferred that the 5A, X0 karyotype observed in *L*. *sigmodontis*, *Acanthocheilonema viteae*, *Loa loa*, *Setaria equina*, and *S*. *digitata* was likely to be the ancestral filarial karyotype (Fig 2A). The NigonX+NigonD fusion therefore occurred in (or prior to) the last common filarial ancestor, which is consistent with the degree of intermixing between the NigonX and NigonD genes observed in the *L*. *sigmodontis* X chromosome.

XY sex chromosome systems have evolved twice independently from X0 systems in filarial nematodes [17] (Fig 2A). In both cases, XY evolution coincided with an X-autosome fusion, but involving two different autosomes [19] (Fig 2A). In the ancestor of *Dirofilaria* and *Onchocerca*, a fusion between NigonE and the X chromosome (NigonX+NigonD) led to a 4A, XY karyotype in *Dirofilaria immitis* (Fig 2B). Consistent with this, the *D*. *immitis* X chromosome (28.2 Mb) is approximately twice the size of the autosomes (which range in size from 14.0 to 15.6 Mb). An additional autosome-autosome fusion (between NigonA and NigonN) occurred in the lineage leading to *O*. *volvulus*, generating its 3A, XY karyotype (Fig 2B). A similar autosome-to-autosome fusion is likely to be present in *Onchocerca gibsoni* [17], which is closely related to *O*. *volvulus* [18]. Other *Onchocerca* species have 4A, XY karyotypes and presumably lack this fusion [17]. The X chromosomes of both *D*. *immitis* and *O*. *volvulus* retain a structure reflecting their origins. The NigonE partition remains distinct from the intermixed NigonX+NigonD partition and the fusion breakpoint retains features of nematode chromosome ends, such as higher repeat content (Figs 2B and S2). We note that the *D*. *immitis* genome was scaffolded on the *O*. *volvulus* assembly [28], and thus that there may be reference bias in the proposed chromosome structure.

In the ancestor of *Brugia* and *Wuchereria*, an independent fusion occurred between NigonN and the X chromosome, generating the 4A, XY karyotype seen in all species in this clade (Fig 2A) [17,18]. The fusion occurred at the opposite end of the X chromosome relative to the *Onchocerca* (and *Dirofilaria*) fusion (S3–S6 Figs). The X chromosome is again substantially larger than the autosomes (24.9 Mb versus 13.4–14.7 Mb) and the fusion strongly patterns the new chromosome. The NigonN partition remains distinct from the NigonX+NigonD partition and the fusion breakpoint is evident in the repeat distribution (Figs 2B and S2). In summary, filarial XY chromosome systems have evolved twice, independently, from an ancestral 5A, X0 karyotype via distinct X-autosome fusion events.

### Filarial Y chromosomes are derived from unfused homologues of fused autosomes

**Fig 3:**
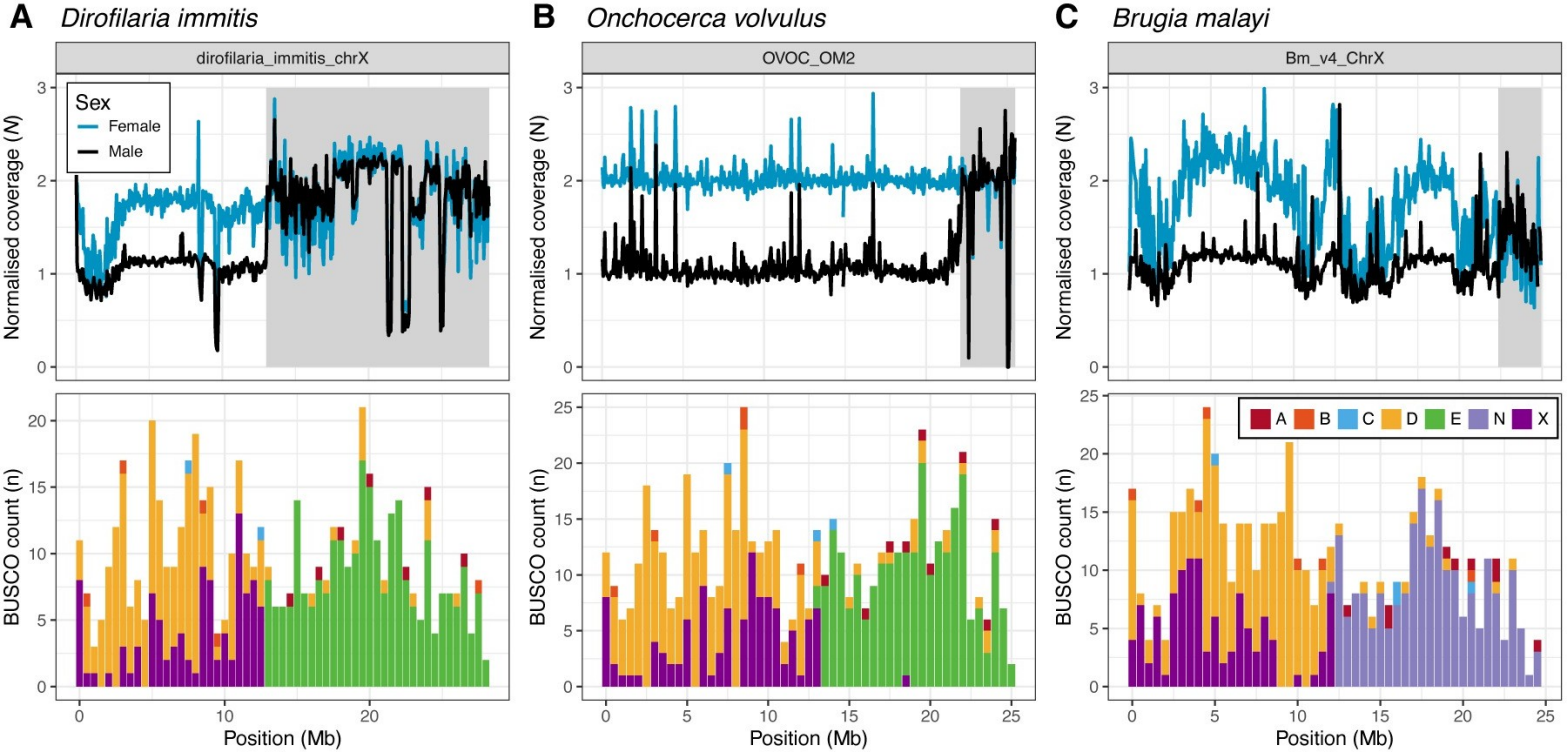
Filarial Y chromosomes are derived from unfused homologues of fused autosomes. Male and female read coverage and Nigon element partitions in the X chromosomes of (A) *D*. *immitis*, (B) *O*. *volvulus*, and (C) *B*. *malayi*. Normalised coverages (*N*) are calculated by dividing the coverage in each 50 kb window by the median autosomal coverage. The histogram of locations of BUSCO loci allocated to Nigon elements (coloured as in Fig 1C) are binned in 500 kb windows. Regions that have diploid coverage in males are shown by grey shading.

Filarial Y chromosomes are hypothesised to be derived from the unfused allelic homologue of an autosome that has undergone an X-autosome fusion [17,19]. Under this model, the ancestrally autosomal portion of the X chromosome (derived from NigonE in *D*. *immitis* and *O*. *volulvus* and NigonN in *B*. *malayi*) should be diploid in males, and may continue to recombine, whereas the ancestrally X-derived portion (NigonX+NigonD) should remain haploid in males. To determine if this is the case, we aligned male- and female-specific short-read data to the *D*. *immitis*, *O*. *volvulus*, and *B*. *malayi* reference genomes and calculated per-base read coverage (Fig 3).

In *D*. *immitis*, the entirety of the NigonE partition of the X chromosome (spanning 15.2 Mb) is diploid in males, while the ancestrally X-derived partition is haploid (Fig 3A). The divergence between the NigonE partition of the X and the Y is low, with an average of 1 SNP every 2,215 bp (S7 Fig and S1 Table). The *D*. *immitis* Y chromosome therefore appears to be an intact, unfused NigonE autosome. In *O*. *volvulus*, the region of the NigonE partition that has diploid coverage is substantially shorter, covering only 3.4 Mb (Fig 3B). The remaining 8.6 Mb of the NigonE-derived sequence has haploid coverage in males, suggesting that the Y chromosome copy of the NigonE element has largely degenerated in *O*. *volvulus*. Based on analyses of the divergence between X and Y in other XY systems, the divergence between X and Y is likely to have been through several “strata” of regions which reflect the progressive but episodic loss of recombination along the former homologues. Consistent with this, SNP density is elevated in *O*. *volvulus* males in the region neighbouring the diploid region (S7 Fig) and there is an average of 1 SNP every 314 bp within the diploid region (S1 Table). In addition, many of the *O*. *volvulus* Y contigs identified by [29] align to the left boundary of the diploid region of the NigonE partition, and show on average 13% nucleotide identity (S8 Fig).

In *B*. *malayi*, there is a 2.6 Mb diploid region, which is found in the NigonN partition of the chromosome (Fig 3C). As in *O*. *volvulus*, a large portion of the NigonN partition is haploid in males, suggesting that the *B*. *malayi* Y chromosome has also undergone degeneration. Heterozygous SNP density is also elevated at the left boundary of the diploid region in males (S7 Fig) and many of the previously identified Y chromosome contigs [20] align to the left of the diploid region (S8 Fig). In summary, the regions shared by the X and Y chromosomes in all three species are ancestrally autosomal, confirming that filarial Y chromosomes are derived from the unfused homologue of the autosome following X-autosome fusion.

## Discussion

We have assembled a chromosome-level reference genome for the filarial nematode *L*. *sigmodontis* and confirmed that it has a karyotype of five autosomes and an X chromosome [17], that the origin of these chromosomes can be explained by the Nigon element model of seven ancestral linkage groups in Rhabditida [19], and that the X chromosome is a relatively old fusion of the NigonX and NigonD elements. We combined these new data with publicly available data for other filarial species to explore the origins of the XX/XY sex chromosome systems of two clades of filarial nematodes that include major parasites of humans (*Onchocerca* plus *Dirofilaria*, and *Brugia* plus *Wuchereria*). We confirm the independent origin of these XX/XY systems through the fusion of different autosomes to the NigonX+NigonD ancestral X chromosome and show that the regions shared by the neo-X and neo-Y chromosomes correspond to ancestrally autosomal sequence in three species with chromosome-level reference genomes.

The X chromosome of nematodes (marked by the loci defining NigonX) is frequently involved in fusion events with autosomes [19]. Many nematode species, including *C*. *elegans*, therefore have XX/X0 sex determination involving X chromosomes that are the product of X-autosome fusion events. The ancestors of these species presumably initially possessed neo-Y chromosomes derived from the unfused homologue of the fused autosomal Nigon elements, but these neo-Y have since been lost. Like filarial XX/XY species, the free-living nematode *Pristionchus exspectatus* has a neo-XY system that originated through a very recent fusion of an ancestral X chromosome (which was solely NigonX) with an autosome (NigonN) [26]. Female *P*. *exspectatus* carry two copies of the fused NigonX+NigonN X chromosome and the male has one NigonX+NigonN X and one NigonN chromosome (the Y). As in filarial nematodes, there are no data indicating that the NigonN neo-Y has any functional role in sex determination. We suggest that sex determination in the filarial and *Pristionchus* XX/XY species is still likely to be functionally mediated via an X-chromosome counting mechanism and that the neo-Y chromosomes play no genetic part in sex determination. The neo-Y chromosomes observed in filarial nematodes and in *P*. *exspectatus* are likely to be intermediates in the transition of the newly X-linked, ancestrally-autosomal chromosome portion from diploidy to haploidy. This transition likely begins with the degeneration of the neo-Y chromosome, which necessitates the spread of the X-chromosome dosage compensation system over the newly sex-linked compartment, and the resolution of any haploinsufficient loci (such as masked deleterious alleles) in the ancestrally autosomal portion of the X chromosome. We note that B chromosomes have been observed in *Onchocerca* species [31,32] and we speculate that these could be fragments of a degenerating Y. Following the spread of dosage compensation, the degenerated Y chromosome is free to be lost.

Degeneration of sex-limited chromosomes (Y or W) is usually ascribed to the suppression of recombination around a sex-determining locus that progressively spreads across the chromosome [5]. Why, then, have the Y chromosomes of *O*. *volvulus* and *B*. *malayi* degenerated in the absence of evident sex-determining loci? The pattern of degeneration of the neo-Y chromosomes in *B*. *malayi* and *O*. *volvulus* may be the result of the patterning of recombination between the neo-X and neo-Y chromosomes. In a *C*. *elegans* strain that had undergone an X-autosome fusion, generating a neo-XY system, the landscape of recombination between the neo-X and neo-Y chromosomes was repatterned such that crossover events were concentrated into a short region of the ancestrally autosomal segment distal to the fusion point [33]. If recombination was repatterned in a similar way after the X-autosome fusions in the ancestors of *B*. *malayi* and *O*. *volvulus*, the relative lack of recombination in the region proximal to the fusion point could drive the observed stepwise pattern of Y chromosome degeneration spreading from the point of fusion. In addition to recombination repatterning, other mechanisms that promote the spread of recombination suppression between sex chromosomes, such as selection for linkage between sexually antagonistic loci or deleterious mutations and the sex-linked regions [5,34–36], may have contributed to the degeneration of the *B*. *malayi* and *O*. *volvulus* Y chromosomes.

It is striking that, in contrast to *O*. *volvulus* and *B*. *malayi*, the neo-Y in *D*. *immitis* shows very little degeneration and retains alignable coverage along the full length of the homologue fused to the X chromosome. While this fusion appears to be homologous to the one in *O*. *volvulus*, under this assumption, the lack of degeneration of the *D*. *immitis* Y suggests that recombination repatterning or the evolution of dosage compensation have followed distinct evolutionary paths in the two lineages. An alternate explanation could be that the *Dirofilaria* and *Onchocerca* fusions are in fact independent, and the *D*. *immitis* Y chromosome is less degenerated because the fusion was more recent. This requires two independent fusions of the ancestral X and NigonE in the same orientation, which is possible but seems unparsimonious if we assume that all autosomes are equally likely to fuse to the X chromosome. Alternatively, we note that the contig-level *D*. *immitis* genome [37] was scaffolded on the *O*. *volvulus* chromosomal assembly [28], and there is a possibility that this reference-guided approach may have categorically biassed the outcome. Without independent chromosomal assembly, characterising the recombination landscapes in both species, or generating chromosome-level reference genomes for other species in this clade, we are not able to distinguish between these possibilities.

The evolution of neo-Y chromosomes is common and often involves X-autosome fusion, for example in *Drosophila* [7,38]. The model proposed for the origin of the Y chromosomes in filarial nematodes and *P*. *exspectatus* [26] means that these nematode Y linkage groups are not evolutionarily homologous. As noted above, XX/XY systems are rare in nematodes, and apart from these examples, there is only a questionable identification of Y-like chromosomes in *Trichuris* species [39,40]. These could also be neo-Y elements and we note that B chromosomes, which we speculate could be fragments of degenerating Y chromosomes, have been described in *Trichuris* species [41]. It is therefore likely that the ancestral nematode possessed an XX/X0 sex chromosome system rather than an XX/XY system, as previously suggested [42], with the XX/XY systems observed in Onchocercidae, *Pristionchus exspectatus*, and *Trichuris* species being evolutionarily derived.

Nematodes show great variability in karyotype, genome organisation, and reproductive modes [19,43,44]. Importantly, this diversity is present within species-rich clades and many transitions have multiple independent occurrences, such as the homoplastic origins of the XX/XY system in filarial nematodes. Coupled with a wealth of new chromosomally-complete genomes, the emerging synthesis of patterns of chromosomal evolution in the phylum promises to become a rich ground to explore larger questions of the drivers of and constraints on evolution. The ancestral linkage groups defined for Rhabditida, the Nigon elements [19], are key organising principles in this endeavour.

## Methods

### Ethics statement

The life cycle of *L*. *sigmodontis* was maintained at the University of Manchester in accordance with the United Kingdom Animals (Scientific Procedures) Act of 1986 under a Project License (70/8548 PP4115856) granted by the UK Home Office and approved by the University of Manchester Animal Welfare and Ethical Review Body.

### Collection of adult *L*. *sigmodontis*

Mongolian jirds (*Meriones unguiculatus*) were infected with 80 infective L3 larvae by intraperitoneal injection; animals were maintained at 27°C and 75% relative humidity. Euthanasia was performed by cervical dislocation 120 days post-infection and adult worms were collected from the peritoneal cavity into PBS. Individual worms of each sex were placed in FluidX tubes chilled on dry ice. Two pools comprising 10 adult males and 10 adult females were collected in the same way. The samples were stored at -80°C.

### DNA extraction and genome sequencing

We extracted DNA from a whole adult female *L*. *sigmodontis* using the New England Biolabs Monarch HMW DNA Extraction Kit for Tissue using the manufacturers’ standard input protocol with the following modifications. Briefly, we transferred the frozen tissue to a 1.5 ml Monarch pestle tube before disruption. We digested the tissue for 30 min at 56°C with 600 rpm before adding RNase A. We eluted the DNA in 200 μl Monarch gDNA Elution Buffer II. The DNA was sheared twice with the Megaruptor 3 from Diagenode with a velocity setting of 30 and 31. The sheared DNA was SPRI cleaned with the ProNex Size-Selective Purification System in a ratio of 1x (v/v) and eluted in 48 μl PacBio Elution buffer. The average DNA fragment size was ~15.8 kb.

A PacBio library was prepared by the Long Read Team of the Scientific Operations core at the Wellcome Sanger Institute using the PacBio Low DNA Input Library Preparation Using SMRTbell Express Template Prep Kit 2.0. The library was sequenced on a single PacBio Sequel IIe flow cell.

Hi-C library preparation and sequencing were performed by the Long Read Team of the Scientific Operations core at the Wellcome Sanger Institute. Two pellets comprising 10 adult males and 10 adult females were processed using the Arima Hi-C version 2 kit following the manufacturer’s instructions. Illumina libraries were prepared using the NEBNext Ultra II DNA Library Prep Kit. Each library was sequenced on one-eighth of a NovaSeq S4 lane using paired-end 150 bp sequencing.

### RNA extraction and sequencing

We independently extracted and sequenced RNA from two female *Litomosoides sigmodontis* individuals. Briefly, we added 800μl TRIzol to each single frozen female and froze the sample in liquid nitrogen, which we then thawed at 37°C in an Eppendorf Thermomixer with 800 rpm. This freeze-thaw cycle was repeated three times. The thawed sample was incubated at room temperature for 10 min before 200 μl Chloroform:Isoamyl alcohol 24:1 was added. The sample was vortexed for 30 sec and incubated for 3 min at room temperature before being centrifuged at 12,000 g for 15 min at 4°C. After centrifugation, we transferred the upper clear phase to a new Eppendorf tube. We added 500 μl Isopropanol and incubated the sample on ice for 10 min. We centrifuged the sample for 10 min, 12,000 g at 4°C. The supernatant was discarded and the pellet was washed two times with 75% Ethanol and centrifuged at 7,500 g for 5 min. We removed the ethanol and allowed the RNA pellet to dry for 5 min. RNA was eluted in 100 μl MagMAX Total RNA Elution Buffer.

RNA library preparation and sequencing were performed by the Scientific Operations core at the Wellcome Sanger Institute. The two RNA extractions were quantified with the QuantiFluor RNA System (Promega), using the Mosquito LV (SPT Labtech), Agilent Bravo and FLUOstar Omega plate reader (BMG Labtech) and normalised to 100 ng in 50 μl using the Tecan Freedom EVO liquid handling platform. Library construction was performed using a custom NEBNext Ultra II RNA kit (New England Biolabs) on an Agilent Bravo NGS workstation. PCR was set-up using 2X KAPA HiFi HotStart ReadyMix (Roche) and custom unique dual indexing primers (IDT) with the following conditions: 98°C 45 sec, 14 cycles of [98°C 15 sec, 65°C 30 sec, 72°C 2 min], 72°C 1 min. PCR amplified libraries were purified using SPRIselect beads (Beckman Coulter) on a Zephyr liquid handling platform (Perkin Elmer). Libraries were quantified with the Accuclear Ultra High Sensitivity dsDNA Quantitation kit (Biotium). Assay setup was performed on a Mosquito LV (SPT Labtech) and Agilent Bravo NGS workstation. Fluorescence was measured on a FLUOstar Omega plate reader (BMG Labtech). Libraries were pooled in equimolar amounts on a BioMek NX-8 (Beckman Coulter). Pooled libraries were quantified on a Bioanalyzer (Agilent). Libraries were normalised to 2.8nM prior to sequencing. Each library was sequenced on a 1/96th of a NovaSeq S4 lane using paired-end 150 bp sequencing.

### Genome assembly

We removed adapter sequences from the PacBio HiFi data using HiFiAdapterFilt [45]. We used Jellyfish 2.3.0 [46] to count *k*-mers of length 31 in each read set and GenomeScope 2.0 [47] to estimate genome size and heterozygosity. We first generated a preliminary assembly of the PacBio HiFi data using hifiasm v0.16.1-r375 [48]. We randomly subsampled 10% of the male Hi-C reads using samtools 1.14 [49] and aligned them to the hifiasm primary assembly using bwa mem 0.7.17-r1188 [50], filtered out PCR duplicates using picard 2.27.1–0 (available at http://broadinstitute.github.io/picard/), and scaffolded the assembly using YaHS [51]. We ran BlobToolKit 2.6.5 [52] on the scaffolded assembly and used the interactive web viewer to manually screen for scaffolds derived from non-target organisms. We identified a single scaffold that was of non-nematode origin that corresponded to the *Wolbachia* endosymbiont genome, which we processed separately (see below). After removing the *Wolbachia* scaffold, we used MitoHiFi 2.2 [53] to extract and annotate the mitochondrial genome. Finally, we removed residual haplotypic duplication from the assembly using purge_dups 1.2.5 [54] and used seqkit [55] and BUSCO 5.2.2 [27] with the nematoda_odb10 lineage to calculate assembly metrics and assess biological completeness. We assessed base-level accuracy and *k*-mer completeness using Merqury 1.3 [56].

### Hi-C scaffolding and manual curation

To generate a chromosome-level reference genome for *L*. *sigmodontis*, we scaffolded the primary assembly using the male Hi-C data as previously described. We manually curated the scaffolded assembly as in [57] and used PretextView 0.2.5 (available at https://github.com/wtsi-hpag/PretextView) to manually inspect the Hi-C signal. Two contigs were identified as haplotypic duplication and were removed from the primary assembly. A final Hi-C contact map was generated using Juicer 2.0 [58] and Juicebox 1.11.08 (available at https://github.com/aidenlab/Juicebox) and is shown in Fig 1A.

### *Wolbachia* genome assembly

The *Wolbachia* contig produced by hifiasm was not circular. To generate a circular *Wolbachia* genome, we aligned the PacBio HiFi reads to the hifiasm contig using minimap2 2.24-r1122 [59] and extracted mapped reads. We assembled the mapped reads using flye 2.9-b1768 [60], which yielded a circular 1.05 Mb contig that shared 100% nucleotide identity with the publicly available circular *w*Ls genome (GCA_013365435.1) [61] and differed in length by just 1 bp. We annotated the genome using Prokka 1.14.6 [62] and rotated the genome to start with HemE, as in [63].

### Gene prediction

Prior to predicting protein-coding genes, we used Earl Grey 2.0 [64] to perform *de novo* repeat identification on the *L*. *sigmodontis* genome. We provided the curated repeat library to RepeatMasker 4.1.2-p1 [65] to soft-mask transposable elements. To predict protein-coding genes, we aligned the two libraries of short-read RNA-seq data to the *L*. *sigmodontis* genome using STAR [66] and provided the resulting BAM file to BRAKER 2.1.6 [67]. We independently predicted genes using BRAKER by supplying the OrthoDB v1 nematoda dataset [68] as protein homology evidence. We used TSEBRA v1.0.3 [69] to combine both sets of predictions using a parameter set that favoured models derived from RNA-seq evidence over those derived from protein homology evidence (weight of 10000 for RNA-seq models and 1 for protein homology models) and which retained all *ab initio* models (i.e. those that did not have support from either RNA-seq or proteins).

### Coverage estimation in *L*. *sigmodontis*

To estimate per-base read coverage of each chromosome in the *L*. *sigmodontis* reference genome, we randomly subsampled 10% of the male and female Hi-C reads using samtools and aligned them to the genome using bwa mem, as previously described. We used BEDtools v2.30.0 [70] to generate non-overlapping windows of various sizes and mosdepth 0.3.3 [71] to calculate coverage in each window.

### Nigon painting

To analyse the distribution of Nigon elements in filarial chromosomes, we ran BUSCO using the nematoda odb10 dataset on chromosome-level reference genomes for *L*. *sigmodontis*, *D*. *immitis* [28], *O*. *volvulus* [29], and *B*. *malayi* [72] (see S2 Table for accessions). We used buscopainter (available at https://github.com/lstevens17/buscopainter) to assign each BUSCO gene to a Nigon element, as defined by [19].

### Filarial phylogeny

To infer a filarial phylogeny, we used busco2fasta (available at https://github.com/lstevens17/busco2fasta) to identify and extract the protein sequences of 1,757 BUSCO genes that were single-copy in the nxLitSigm11.1 reference genomes and genomes of ten other filarial species (S2 Table). We aligned the protein sequences using FSA 1.15.9 [73] and concatenated the alignments of each single-copy orthogroup into a supermatrix using catfasta2phyml v1.1.0 (available at https://github.com/nylander/catfasta2phyml). We used IQ-TREE 2.2.0.3 [74] to infer the filarial phylogeny under the LG substitution model [75] with gamma-distributed rate variation among sites. We visualised the resulting species tree using the iTOL web server [76].

### Repeat distribution

To analyse repeat distribution across each chromosome, we used Red 2.0 [77] with a *k-*mer length of 13 to identify repetitive sequences in the *L*. *sigmodontis*, *D*. *immitis*, *O*. *volvulus*, and, *B*. *malayi* genomes. We calculated repeat density in non-overlapping 200 kb windows using BEDtools v2.30.0. We note that the repetitive proportion estimated for *L*. *sigmodontis* by Red (44%) is substantially higher than estimated using Earl Grey (6%), which is likely due to Earl Grey being designed to identify full-length transposable elements, whereas Red identifies repetitive sequences of any size, the majority of which are not transposable elements.

### Inferring the regions shared between the X and Y chromosomes

To define regions that were shared by the X and Y chromosomes of *D*. *immitis*, *O*. *volvulus*, and *B*. *malayi*, we mapped a set of male- and female-derived paired-end short-read Illumina reads (see S3 Table for accessions) to each genome using bwa mem and calculated coverage in non-overlapping 100 kb windows, as previously described. We used a similar definition to [20] to identify regions shared by the X and Y chromosomes: large (> 1 Mb in length), semi-contiguous regions of the X chromosome where no bins were female-dominated (male-to-female coverage ratio < 0.8). To do this, we normalised male and female read coverage by dividing the coverage in each 100 kb window by the autosomal median coverage (the median of all 100 kb windows excluding those on the X chromosomes). We then merged all non-female-dominated bins that were separated by 200 kb or less using BEDtools and retained only windows that were at least 1 Mb in size. Using this approach, we identified a single region that was diploid in males in *B*. *malayi* (Bm_v4_ChrX:22,300,000–24,943,668) and in *O*. *volvulus* (OVOC_OM2:22,100,000–25,485,961). In *D*. *immitis*, there were two non-overlapping windows larger than 1 Mb, but one of these was a 1.4 Mb region that showed low coverage in both males and females (dirofilaria_immitis_chrX:700,000–2,100,000), whereas the other was a 15.2 Mb region that had diploid coverage in both males and females (dirofilaria_immitis_chrX:13,000,000–28,232,375) (Fig 3A).

### Estimating divergence between X and Y chromosomes using variant calling

To estimate the divergence between the X and Y chromosomes of *D*. *immitis*, *O*. *volvulus*, and *B*. *malayi*, we used the BAM files generated previously to call variants in each read set using DeepVariant 1.4.0 [78]. We filtered out any variant labelled with ‘RefCall’ and used BCFtools 1.15 [49] to filter the resulting VCF files to contain only heterozygous biallelic single nucleotide polymorphisms (SNPs). For each read set, we calculated heterozygous SNP density across the genome using 50 kb, non-overlapping windows using BEDtools. To plot the average divergence between the X and Y chromosomes, we inferred the sex of each individual based on the coverage of the X chromosome (S3 Table) and calculated the mean SNP density in each 50 kb window for all male read sets using BEDtools. We also calculated the average SNP density for each male across the entirety of the diploid regions inferred previously (S1 Table).

### Comparing divergence between Y contigs

We removed the 63 *B*. *malayi* Y contigs identified by [20] and 148 *O*. *volvulus* Y contig identified by [29] from each reference genome and then aligned them to the remaining contigs/scaffolds using NUCmer, which is part of the MUMmer package 3.1 [79]. We retained only one-to-one alignments (using delta-filter, which is also part of the MUMmer package) that were 1 kb or longer. We calculated alignment coverage in non-overlapping 50 kb windows using BEDtools, as previously described. We calculated the mean alignment identity by multiplying the identity of each alignment by its length and dividing by the total length of all alignments.

## Supporting information

S1 Fig Telomeric repeat sequence in the *Litomosoides sigmodontis* nxLitSigm11.1 reference genome. Counts of the nematode telomeric repeat sequence (TTAGGC) in 1 kb windows in the nxLitSigm11.1 reference genome.
(TIF)

S2 Fig Repeat distributions in the genomes of four filarial nematode species. Repeat and Nigon element distributions in the genomes of (A) *L*. *sigmodontis*, (B) *D*. *immitis*, (C) *O*. *volvulus*, and (D) *B*. *malayi*. Repetitive sequences were identified using Red with a *k-*mer length of 13 and repeat densities were calculated in 200 kb, non-overlapping windows. Lines represent LOESS smoothing functions fitted to the data. Distribution of counts of BUSCO genes in 500 kb windows in the six *L*. *sigmodontis* chromosomes by their allocation to the seven Nigon elements (coloured as in Fig 1C). The repetitive proportion estimated for *L*. *sigmodontis* genome by Red (44%) is substantially higher than estimated using Earl Grey (6%), which is likely due to Earl Grey being designed to identify full-length transposable elements, whereas Red identifies repetitive sequences of any size, the majority of which are not transposable elements.
(TIF)

S3 Fig Synteny between *D*. *immitis* and *L*. *sigmodontis*. The relative position of 1,979 BUSCO genes in the *D*. *immitis* and *L*. *sigmodontis* genomes. BUSCO genes are coloured by their Nigon assignment. *D*. *immitis* chromosomes are ordered as in Fig 2B.
(TIF)

S4 Fig Synteny between *O*. *volvulus* and *L*. *sigmodontis*. The relative position of 2,198 BUSCO genes in the *O*. *volvulus* and *L*. *sigmodontis* genomes. BUSCO genes are coloured by their Nigon assignment. *O*. *volvulus* chromosomes are ordered as in Fig 2B.
(TIF)

S5 Fig Synteny between *B*. *malayi* and *L*. *sigmodontis*. The relative position of 2,209 BUSCO genes in the *B*. *malayi* and *L*. *sigmodontis* genomes. BUSCO genes are coloured by their Nigon assignment. *B*. *malayi* chromosomes are ordered as in Fig 2B.
(TIF)

S6 Fig Synteny between the X chromosomes of *D*. *immitis*, *O*. *volvulus*, *B*. *malayi* and *L*. *sigmodontis*. The position of BUSCO genes on the X chromosomes of (A) *D*. *immitis*, (B) *O*. *volvulus*, and (C) *B*. *malayi* relative to their position in the *L*. *sigmodontis* genome. BUSCO genes are coloured by their Nigon assignment.
(TIF)

S7 Fig Divergence between filarial X and Y chromosomes. Mean male SNP density and Nigon element partitions in the X chromosomes of (A) *D*. *immitis*, (B) *O*. *volvulus*, and (C) *B*. *malayi*. Lines represent mean heterozygous SNP density in each 50 kb window using all male datasets for each species (S3 Table). Regions that are diploid in males are shown by grey shading. The histogram of locations of BUSCO loci allocated to Nigon elements (coloured as in Fig 1C) are binned in 500 kb windows.
(TIF)

S8 Fig Y contig alignment coverage in the *O*. *volvulus* and *B*. *malayi* X chromosomes. Alignment coverage in 50 kb windows of the 148 *O*. *volvulus* Y contig identified by (Cotton *et al*. 2016) and 63 *B*. *malayi* Y contigs identified by (Foster *et al*. 2020) to the X chromosomes of (A) *O*. *volvulus* and (B) *B*. *malayi*. Regions that are diploid in males are shown by grey shading. Only one-to-one alignments that were 1 kb or longer were considered. The histogram of locations of BUSCO loci allocated to Nigon elements (coloured as in Fig 1C) are binned in 500 kb windows.
(TIF)

S1 Table SNP density in X chromosome regions that are diploid in males. (XLSX)

S2 Table Accessions of genomes used to infer the filarial phylogeny. (XLSX)

S3 Table Accessions of read data used to infer the X chromosome regions that are diploid in males. (XLSX)

S1 Text Comparison of the Tandonnet *et al*. and Gonzalez *et al*. definitions of Nigon elements to those of Foster *et al*. (DOCX)
