# Supplementary figures and images for: The genome of Litomosoides sigmodontis illuminates the origins of Y chromosomes in filarial nematodes
Source: PLoS Genet. 2024 Jan 16;20(1):e1011116. doi: 10.1371/journal.pgen.1011116 (PMC10817185; doi:10.1371/journal.pgen.1011116)

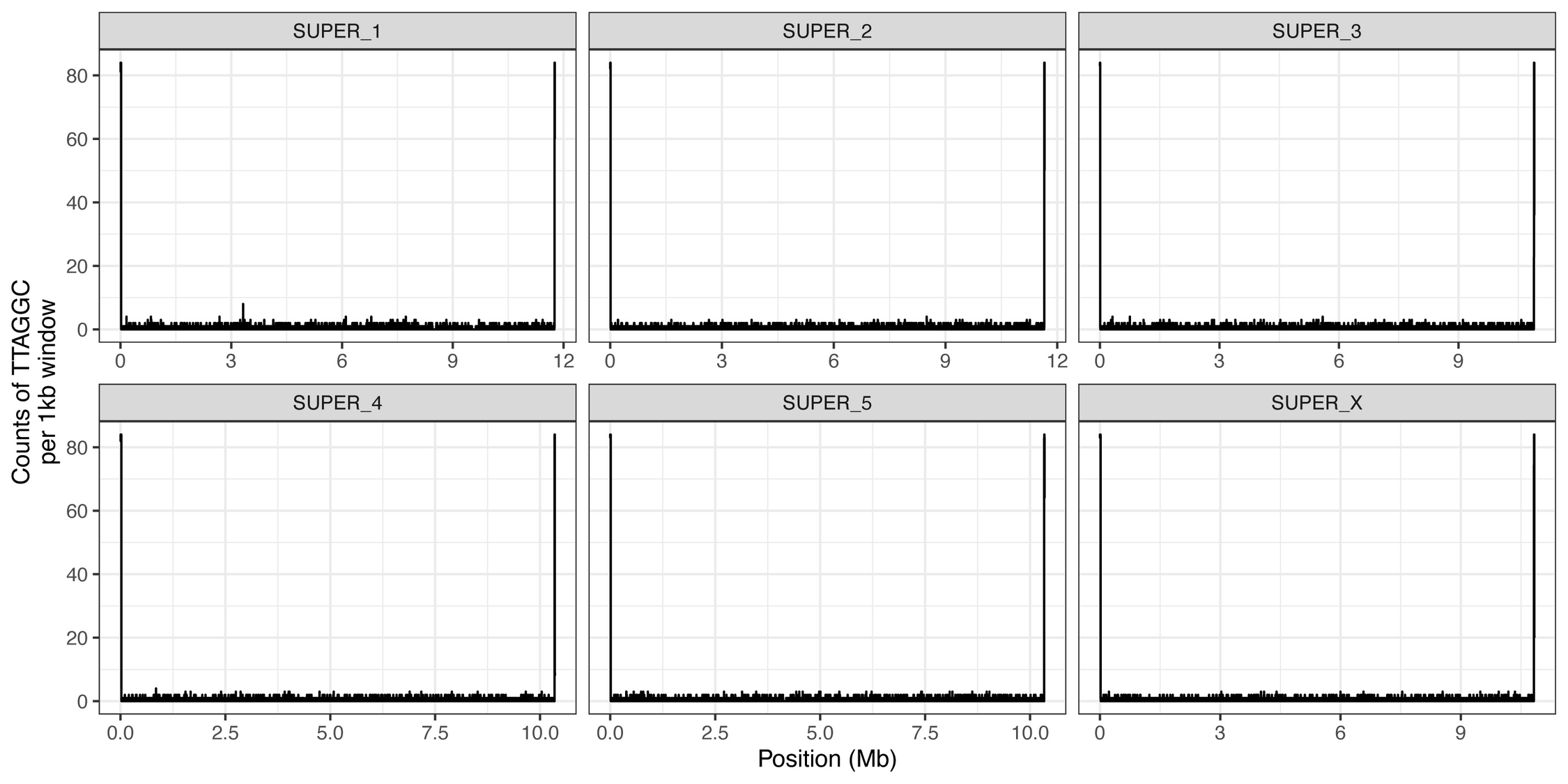

Supplement: S1 Fig — Counts of the nematode telomeric repeat sequence (TTAGGC) in 1 kb windows in the nxLitSigm11.1 reference genome. (TIF) [file pgen.1011116.s001.tif]

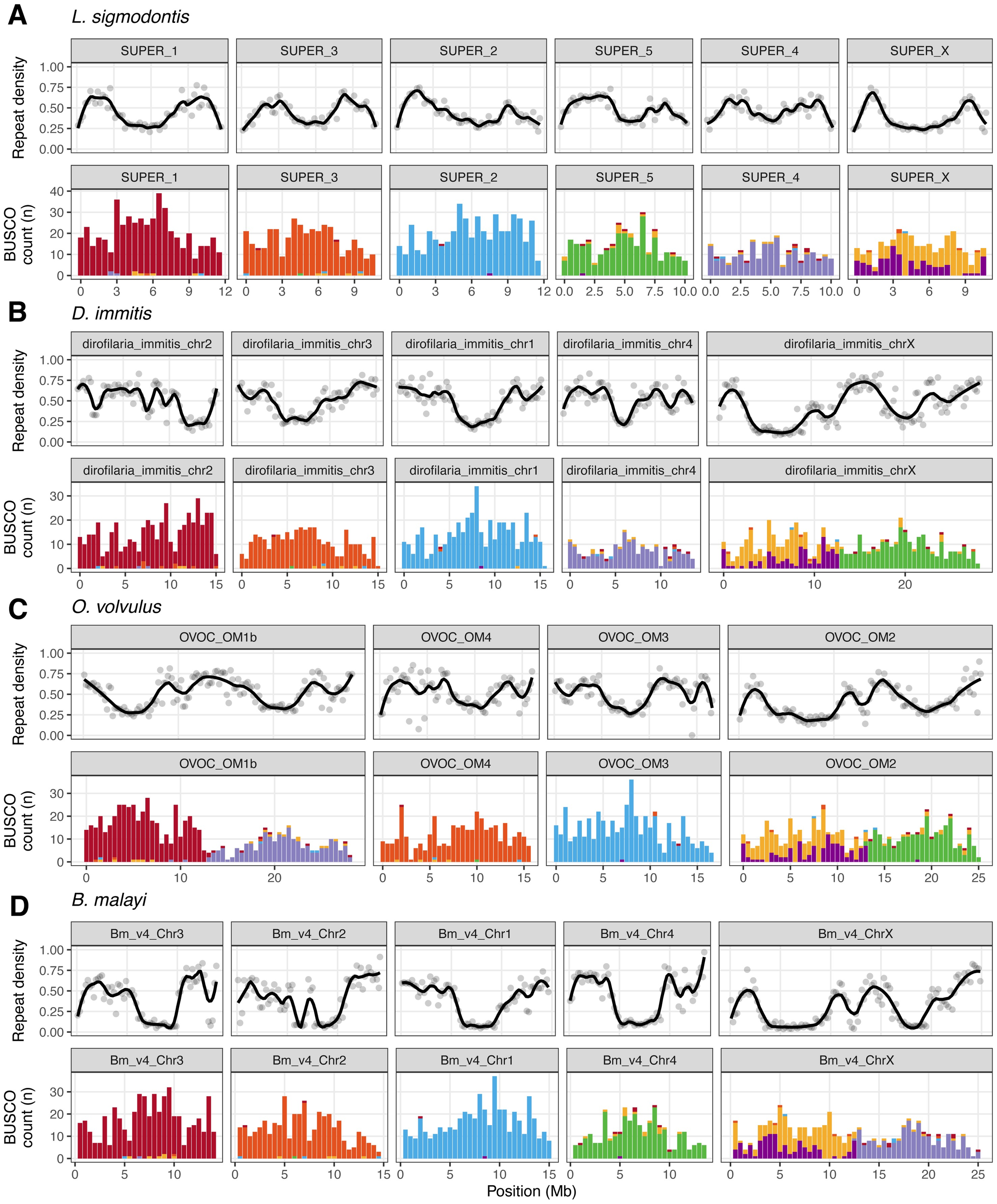

Supplement: S2 Fig — Repeat and Nigon element distributions in the genomes of (A) L. sigmodontis, (B) D. immitis, (C) O. volvulus, and (D) B. malayi. Repetitive sequences were identified using Red with a k-mer length of 13 and repeat densities were calculated in 200 kb, non-overlapping windows. Lines represent LOESS smoothing functions fitted to the data. Distribution of counts of BUSCO genes in 500 kb windows in the six L. sigmodontis chromosomes by their allocation to the seven Nigon elements (coloured as in Fig 1C). The repetitive proportion estimated for L. sigmodontis genome by Red (44%) is substantially higher than estimated using Earl Grey (6%), which is likely due to Earl Grey being designed to identify full-length transposable elements, whereas Red identifies repetitive sequences of any size, the majority of which are not transposable elements. (TIF) [file pgen.1011116.s002.tif]

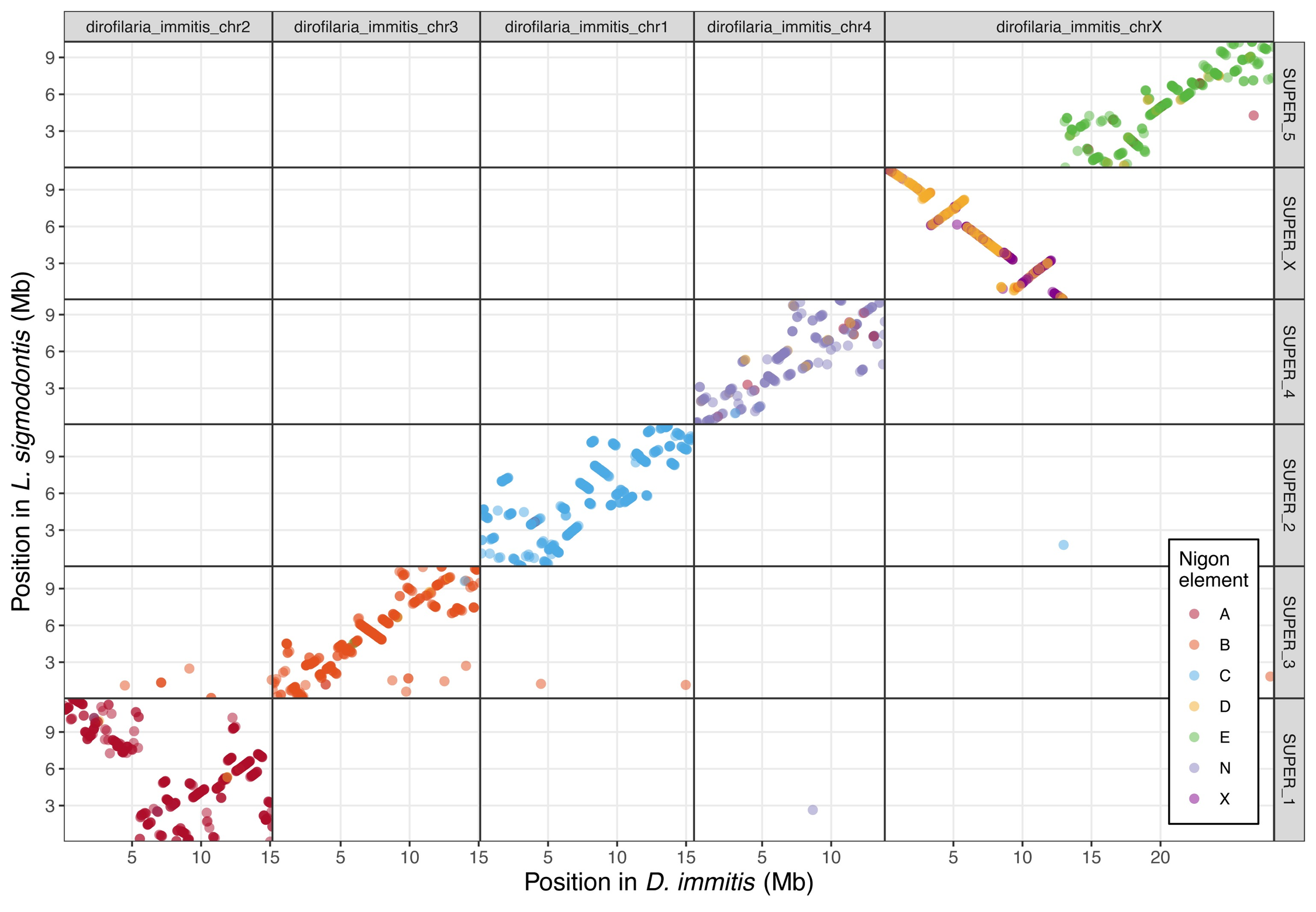

Supplement: S3 Fig — The relative position of 1,979 BUSCO genes in the D. immitis and L. sigmodontis genomes. BUSCO genes are coloured by their Nigon assignment. D. immitis chromosomes are ordered as in Fig 2B. (TIF) [file pgen.1011116.s003.tif]

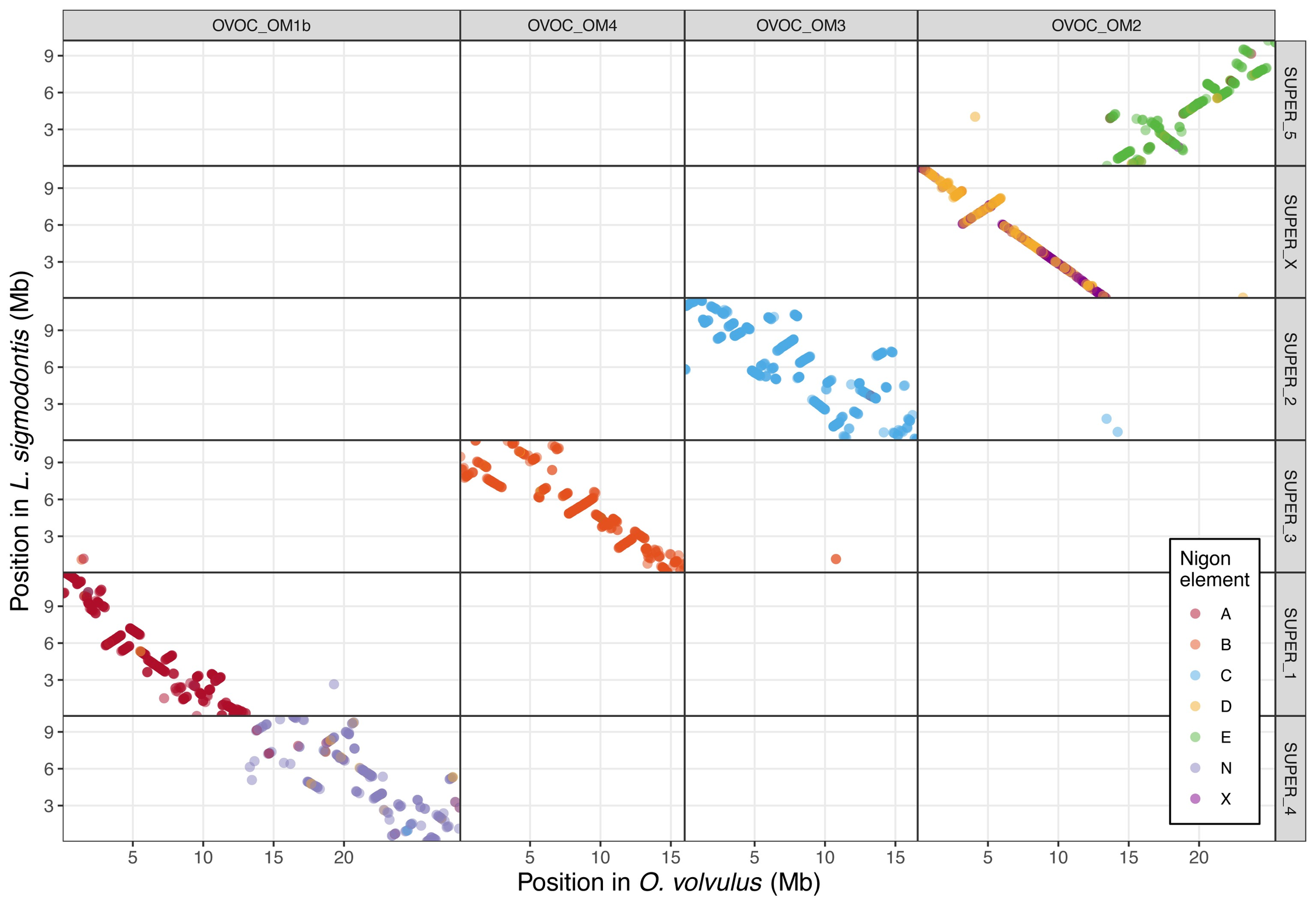

Supplement: S4 Fig — The relative position of 2,198 BUSCO genes in the O. volvulus and L. sigmodontis genomes. BUSCO genes are coloured by their Nigon assignment. O. volvulus chromosomes are ordered as in Fig 2B. (TIF) [file pgen.1011116.s004.tif]

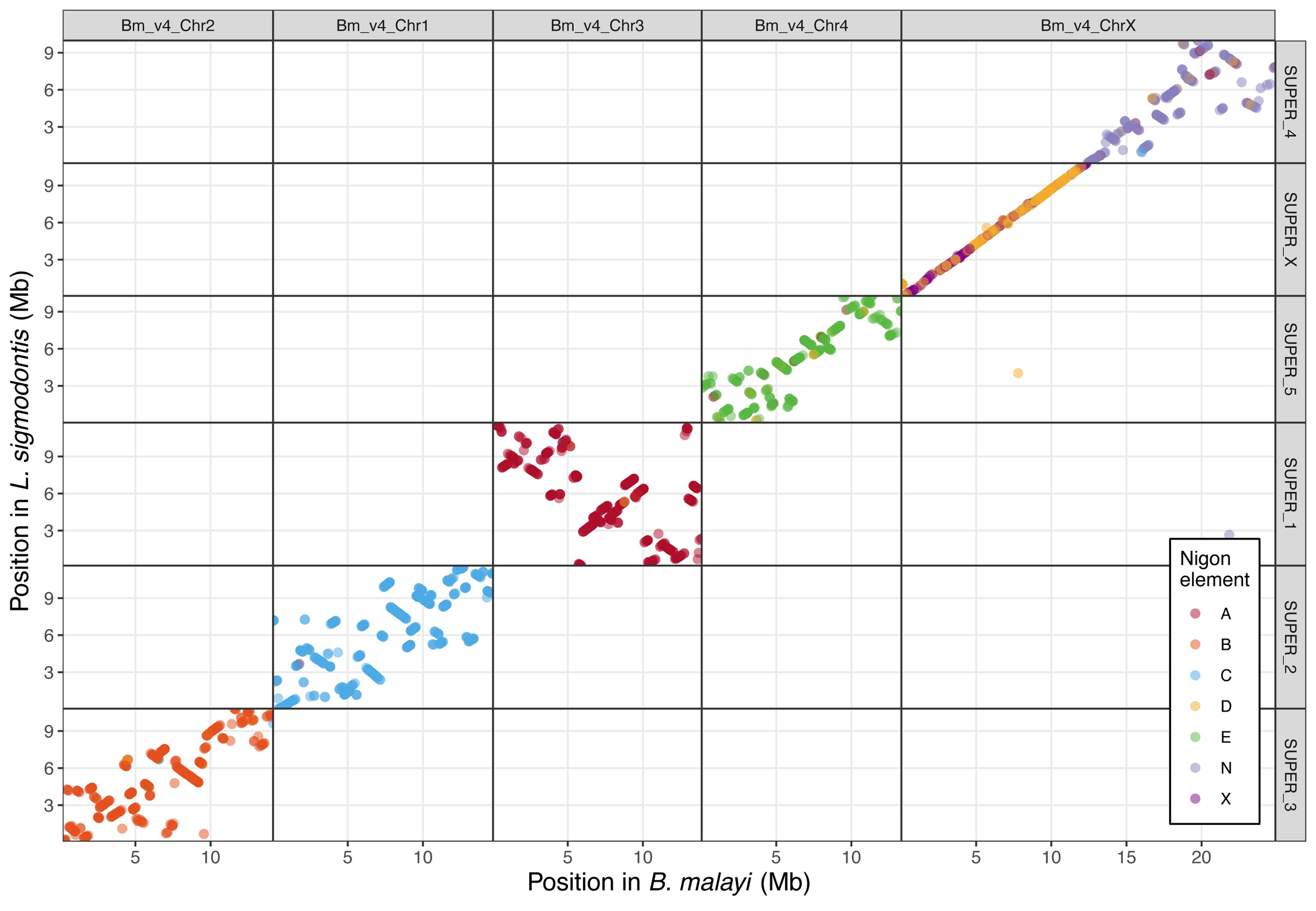

Supplement: S5 Fig — The relative position of 2,209 BUSCO genes in the B. malayi and L. sigmodontis genomes. BUSCO genes are coloured by their Nigon assignment. B. malayi chromosomes are ordered as in Fig 2B. (TIF) [file pgen.1011116.s005.tif]

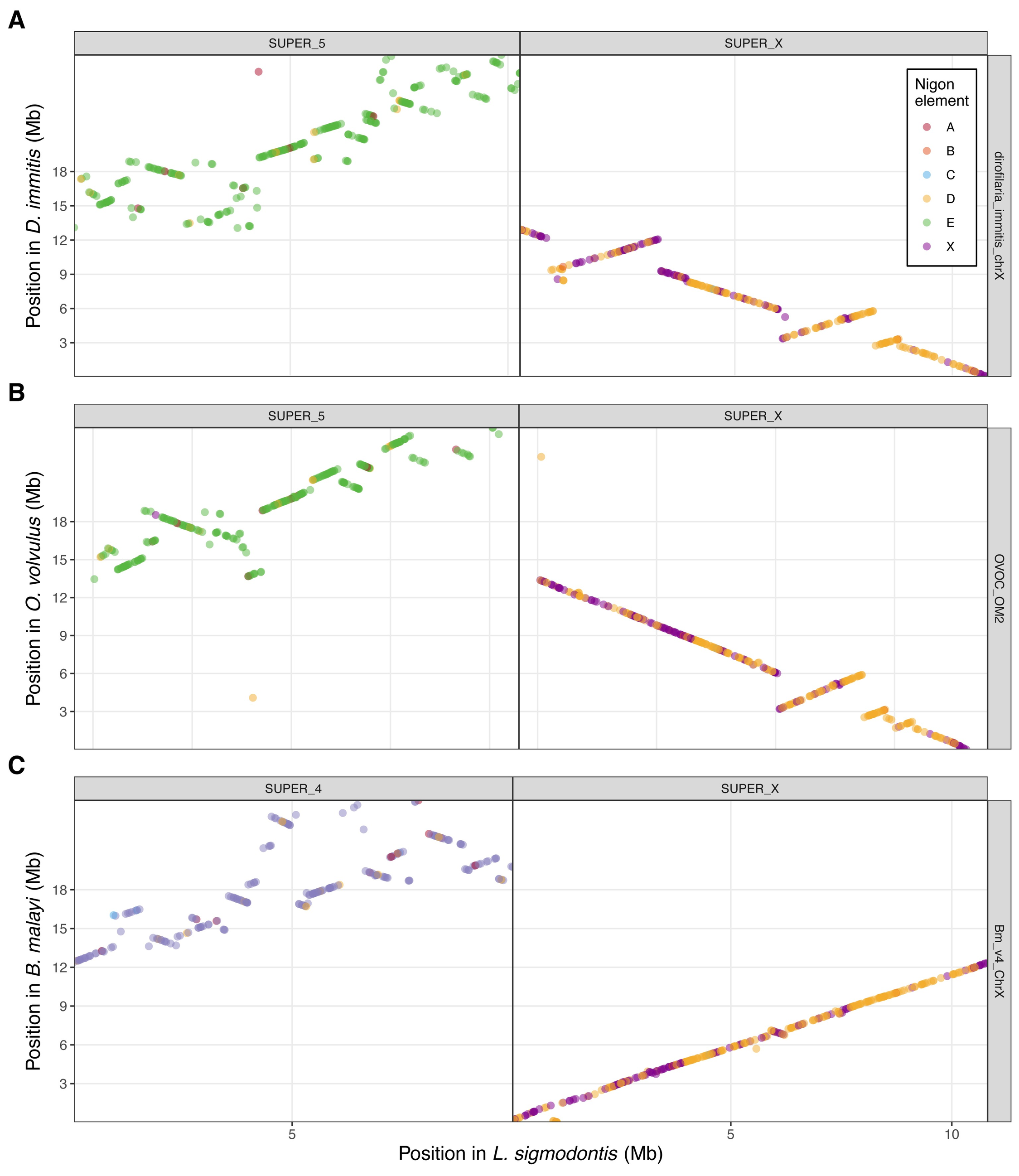

Supplement: S6 Fig — The position of BUSCO genes on the X chromosomes of (A) D. immitis, (B) O. volvulus, and (C) B. malayi relative to their position in the L. sigmodontis genome. BUSCO genes are coloured by their Nigon assignment. (TIF) [file pgen.1011116.s006.tif]

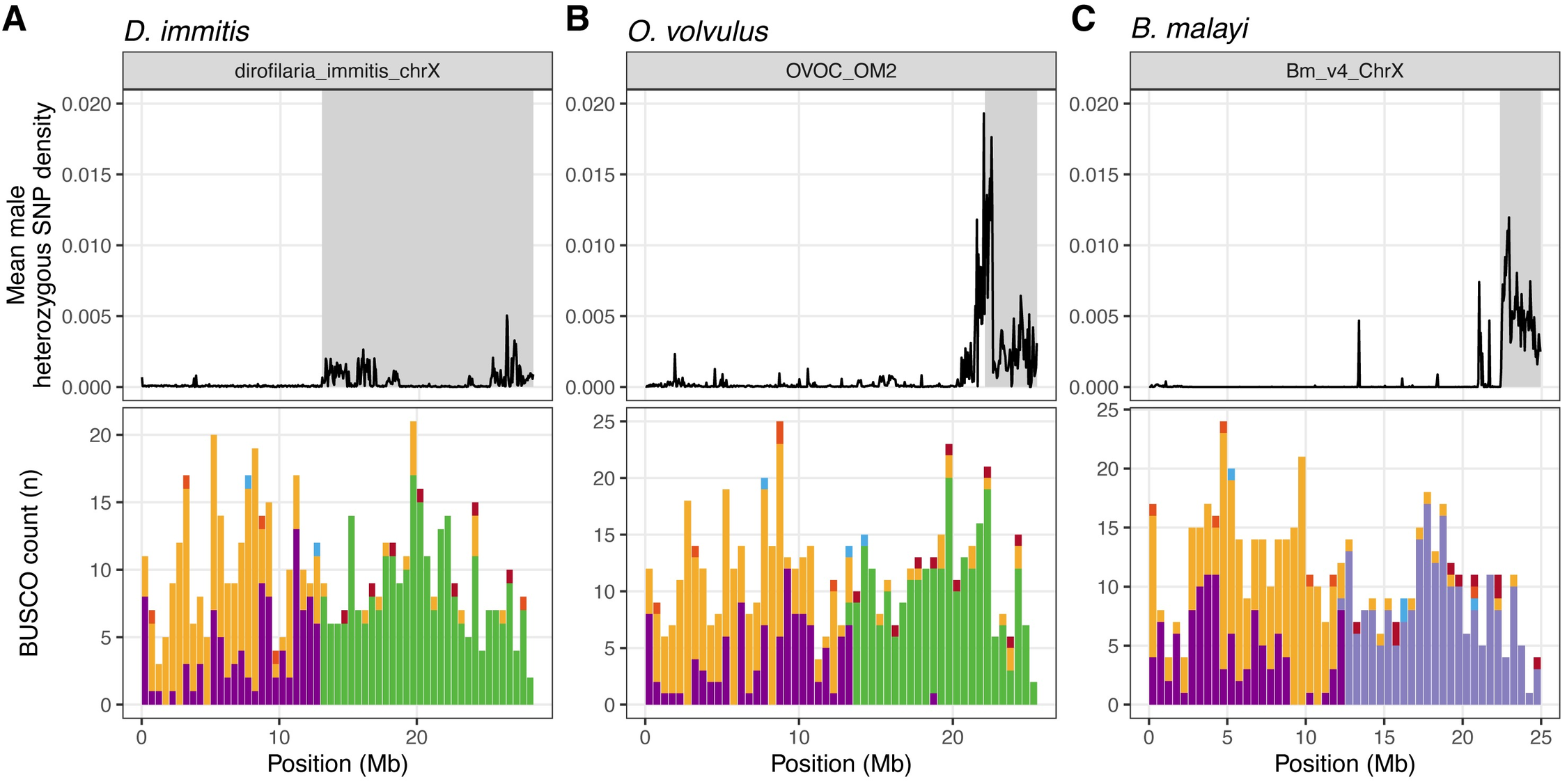

Supplement: S7 Fig — Mean male SNP density and Nigon element partitions in the X chromosomes of (A) D. immitis, (B) O. volvulus, and (C) B. malayi. Lines represent mean heterozygous SNP density in each 50 kb window using all male datasets for each species (S3 Table). Regions that are diploid in males are shown by grey shading. The histogram of locations of BUSCO loci allocated to Nigon elements (coloured as in Fig 1C) are binned in 500 kb windows. (TIF) [file pgen.1011116.s007.tif]

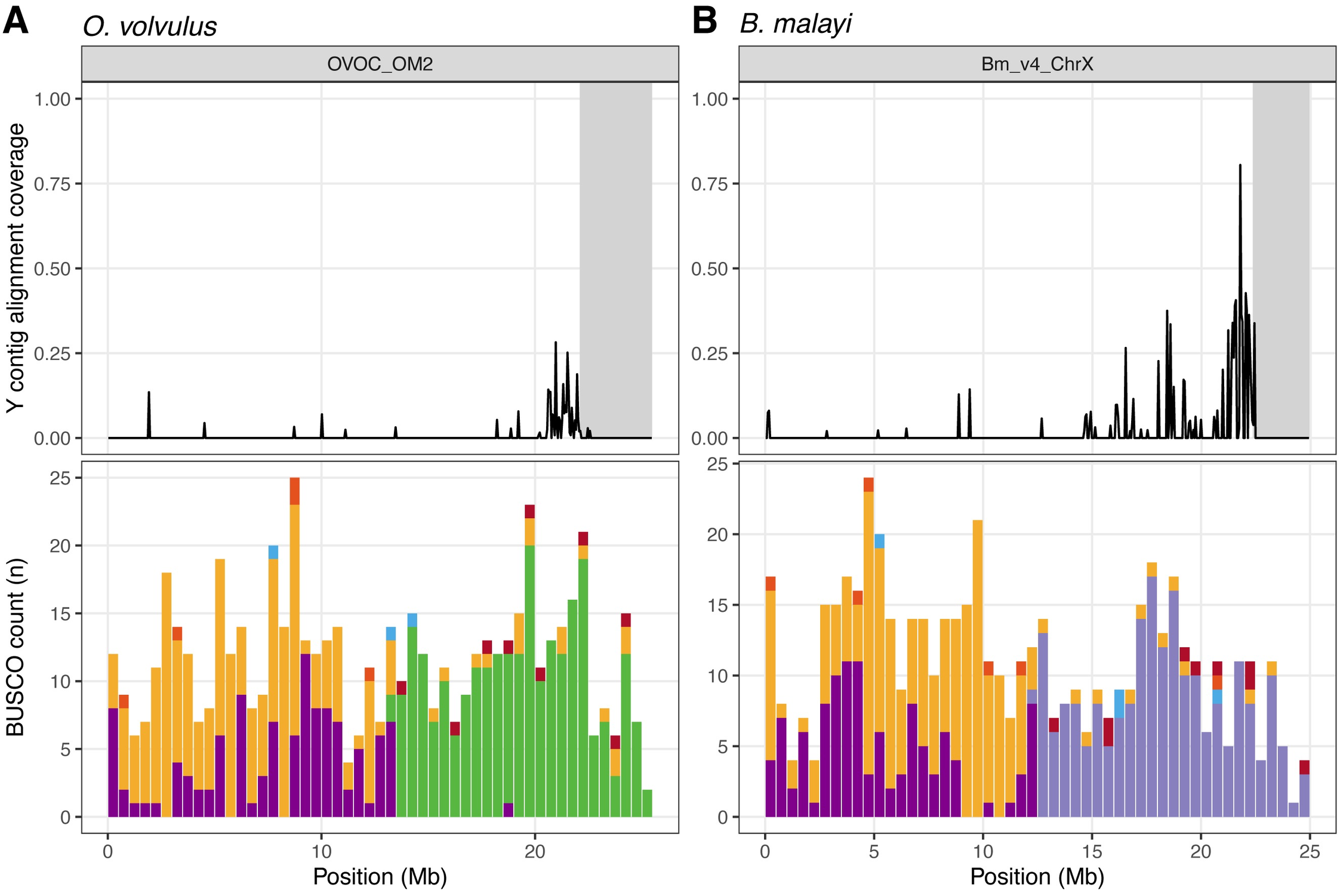

Supplement: S8 Fig — Alignment coverage in 50 kb windows of the 148 O. volvulus Y contig identified by (Cotton et al. 2016) and 63 B. malayi Y contigs identified by (Foster et al. 2020) to the X chromosomes of (A) O. volvulus and (B) B. malayi. Regions that are diploid in males are shown by grey shading. Only one-to-one alignments that were 1 kb or longer were considered. The histogram of locations of BUSCO loci allocated to Nigon elements (coloured as in Fig 1C) are binned in 500 kb windows. (TIF) [file pgen.1011116.s008.tif]
