# Supplementary material for: The genome of Litomosoides sigmodontis illuminates the origins of Y chromosomes in filarial nematodes
Source: PLoS Genet. 2024 Jan 16;20(1):e1011116. doi: 10.1371/journal.pgen.1011116 (PMC10817185; doi:10.1371/journal.pgen.1011116)
Supplement: S1 Text — (DOCX) [file pgen.1011116.s012.docx]

## **S1 text: Comparison of the Tandonnet *et al. [4]* and Gonzalez *et al. [2]* definitions of Nigon elements to those of Foster *et al. [1]***

The seven ancestral rhabditid linkage groups described by Tandonet *et al.* (2019) [4] and Gonzalez de la Rosa *et al.* (2021) [2] were rejected by Foster *et al.* (2020) [1], who found only six. Confusingly, Foster *et al.[1]* used the same naming (“Nigon elements”) and some of the same designation (A-E,X) as Tandonnet *et al.* [4] and Gonzalez de la Rosa *et al. [2].* Below, we discuss the differences between the original description and definition of Nigon elements by Tandonnet *et al.* [4] and Gonzalez de la Rosa *et al.* [2](termed ‘Nigons’ below), and the revised version proposed by Foster *et al.* [1](termed ‘F-Nigons’ below) and show that F-Nigons do not parsimoniously describe rhabditid or filarial chromosome evolution.

Foster *et al.* [1] analysed a small subset (three) of the then-available chromosome-level nematode reference genomes and presented a new set of six F-Nigon elements (Table A), inferred through manual interpretation of Circos plots displaying pairwise PROmer alignments between *C. elegans, B. malayi* and *O. volvulus*. The genomes of *B. malayi* and *O.volvulus* have largely conserved linkage groups apart from the different fusions between the ancestral filarial X chromosome and two different autosomes. These fusions are evident because the ancestrally autosomal and X segments form distinct partitions. Foster *et al.* inferred that the *B. malayi* Y chromosome is orthologous to the X chromosome of *C. elegans* (F-NigonX) and that the *B. malayi* X chromosome is the product of a fusion of F-NigonX with F-NigonD. In addition, they inferred that *O. volvulus* Y is orthologous to *C. elegans* chromosome V (F-NigonE) and that the *O. volvulus* X is formed through a fusion of F-NigonE with F-NigonD. Foster *et al.* [1] note that, in *P. pacificus*, F-NigonX is split between the X chromosome and chromosome I, the latter of which is a fusion of part of F-NigonX with F-NigonE.

As Foster *et al. [1]* did not include the *P. pacificus* reference genome (or chromosomal-level reference genomes for several other nematode species) when inferring F-Nigons, they were not able to detect the fact that the part of F-NigonX that is fused with F-NigonE in *P. pacificus* behaves as a distinct element in filarial nematodes and other species. These loci are in distinct chromosome partitions in *B. malayi* (fused with F-NigonD) and in *O. volvulus* (fused with F-NigonA) and behave independently in other rhabditid genomes (see Gonzalez de la Rosa *et al.[2]*). This set of genes has thus had a coherent and independent history in several different lineages of nematodes. The Foster *et al. [1]* model requires that this subset of genes independently assorted itself from the other F-NigonX loci independently in species separated by tens to hundreds of millions of years of evolution, which is not parsimonious.

Foster *et al.* [1] misidentified the F-NigonD element (equivalent in their model to *C. elegans* chromosome IV) as being the ancestrally sex-linked element in rhabditid nematodes, missing the fact that the F-NigonD element they inferred includes loci found on the X chromosome in *C. elegans* and *P. pacificus.* Foster *et al.* [1] thus had to postulate the conversion of an autosome to a sex chromosome (and vice versa) in clade V nematodes (which includes *C. elegans* and *P. pacificus*). We compare the F-Nigon element definitions to the Tandonnet *et al.* [4] and Gonzalez de la Rosa *et al.* [2] definitions in Table A.

In contrast, the original Nigon model accurately and parsimoniously describes the pattern of orthologue distribution on chromosomes across rhabditid nematode species. Nigons were initially defined in a manual manner similar to that deployed by Foster *et al.* (see Tandonnet *et al. [4]*) but using a larger number of reference genomes. Gonzalez de la Rosa *et al.* [2] revisited the definition of ancestral linkage groups using algorithmic approaches across 14 genomes. In brief, Gonzalez *et al.* [2] defined a set of conserved single-copy orthologues across the analysed species, and described the chromosomal positioning of each orthologue as a vector of chromosomal assignments. Decomposing this vector identified seven clusters of genes, corresponding to Nigons A, B, C D, E, N and X (Table A). The elements defined matched the Tandonnet *et al. [4]* model and the same seven elements have also been recovered using the ALG inference tool syngraph [(Mackintosh et al. 2023)](https://paperpile.com/c/0dTuhV/pt5u) [3].

In contrast to Foster *et al.*, the Tandonnet *et al.* and Gonzalez de la Rosa *et al.* analyses identified the *C. elegans* X chromosome as being the product of a fusion of two distinct ancestral units (NigonX and NigonN) that have since become fully intermixed [1,2,4]. NigonN was observed as an independent linkage group in several analysed species (e.g. *Bursaphelenchus xylophilus*) or was fused to other Nigon elements (e.g. NigonE in *P. pacificus*). Under the Nigon model, genes that define NigonX are always found on the X chromosomes of rhabditid nematodes. The ancestral filarial X chromosome is an old fusion between NigonX and NigonD that predates the diversification of the species analysed here. The autosomes that fuse with the filarial NigonX+NigonD X chromosome are NigonE (*O. volvulus*, *D. immitis*) or NigonN. NigonN is an independent chromosome in *D. immitis* and has fused with NigonA in *O. volvulus* (Fig 2).

#### **Table A. Tandonnet *et al.* and Gonzalez *et al.* definitions of Nigon elements compared to the Foster *et al.* schema. [4,2,1]**

| **Nigon** | **F-Nigon** | **Comment comparing Nigon and F-Nigon assignments** |
| --- | --- | --- |
| **A** | A | Equivalent |
| **B** | B | Equivalent |
| **C** | C | Equivalent |
| **E** | E | Equivalent |
| **D** | D | NigonD is included fully in F-NigonD. F-NigonD additionally includes NigonX in some but not all representations. |
| **N** | X | NigonN is included fully in F-NigonX. F-NigonX additionally includes NigonX in some but not all representations. |
| **X** | Sometimes D, sometimes X | NigonX is included in F-NigonD in filarial nematodes and in F-NigonX in *C. elegans* and *P. pacificus*. In contrast, it is split out as a distinct part of F-NigonX in *P. pacificus*, forming the X chromosome. |

### **References**

1. [Foster, Jeremy M., Alexandra Grote, John Mattick, Alan Tracey, Yu-Chih Tsai, Matthew Chung, James A. Cotton, et al. 2020. “Sex Chromosome Evolution in Parasitic Nematodes of Humans.” *Nature Communications* 11 (1): 1964.](http://paperpile.com/b/0dTuhV/J7ht)
2. [Gonzalez de la Rosa, Pablo Manuel, Marian Thomson, Urmi Trivedi, Alan Tracey, Sophie Tandonnet, and Mark Blaxter. 2021. “A Telomere-to-Telomere Assembly of Oscheius Tipulae and the Evolution of Rhabditid Nematode Chromosomes.” *G3*  11 (1). https://doi.org/](http://paperpile.com/b/0dTuhV/n4MI)[10.1093/g3journal/jkaa020](http://dx.doi.org/10.1093/g3journal/jkaa020)[.](http://paperpile.com/b/0dTuhV/n4MI)
3. [Mackintosh, Alexander, Pablo Manuel Gonzalez de la Rosa, Simon H. Martin, Konrad Lohse, and Dominik R. Laetsch. 2023. “Inferring Inter-Chromosomal Rearrangements and Ancestral Linkage Groups from Synteny.” *bioRxiv*. https://doi.org/](http://paperpile.com/b/0dTuhV/pt5u)[10.1101/2023.09.17.558111](http://dx.doi.org/10.1101/2023.09.17.558111)[.](http://paperpile.com/b/0dTuhV/pt5u)
4. [Tandonnet, Sophie, Georgios D. Koutsovoulos, Sally Adams, Delphine Cloarec, Manish Parihar, Mark L. Blaxter, and Andre Pires-daSilva. 2019. “Chromosome-Wide Evolution and Sex Determination in the Three-Sexed Nematode Auanema Rhodensis.” *G3*  9 (4): 1211–30.](http://paperpile.com/b/0dTuhV/BmjB)
